# Mobile phone thermography of the toes in patients with systemic sclerosis—a pilot study

**DOI:** 10.1093/rap/rkae068

**Published:** 2024-05-22

**Authors:** Ruey Lim, Graham Dinsdale, Joanne Manning, Calvin Heal, Andrea Murray, Ariane L Herrick

**Affiliations:** Centre for Musculoskeletal Research, The University of Manchester, Northern Care Alliance NHS Foundation Trust, Manchester Academic Health Science Centre, Manchester, UK; Salford Care Organisation, Northern Care Alliance NHS Foundation Trust, Salford, UK; Salford Care Organisation, Northern Care Alliance NHS Foundation Trust, Salford, UK; Centre for Biostatistics, The University of Manchester, Manchester, UK; Centre for Musculoskeletal Research, The University of Manchester, Northern Care Alliance NHS Foundation Trust, Manchester Academic Health Science Centre, Manchester, UK; Centre for Musculoskeletal Research, The University of Manchester, Northern Care Alliance NHS Foundation Trust, Manchester Academic Health Science Centre, Manchester, UK; Salford Care Organisation, Northern Care Alliance NHS Foundation Trust, Salford, UK

**Keywords:** feet, Raynaud’s phenomenon, systemic sclerosis, thermography

## Abstract

**Objectives:**

To investigate the hypotheses that in patients with SSc, the temperature gradient between the dorsum of the foot and toes (distal–dorsal difference [DDD]) is ‘more negative’ (toes cooler) than in healthy controls, is greatest along the first (great) toe and that the severities of thermographic abnormalities in the feet and hands are correlated.

**Methods:**

Thermographic images of the dorsum of each hand and foot were captured using a thermal camera attached to an iPhone in 40 patients with SSc and 20 healthy controls. DDDs along the fingers (index, middle, ring and little) and toes (great toe and ‘others’) were measured.

**Results:**

There was a non-significant trend for the great toes to be colder in patients with SSc than in controls. The mean great toe DDD was more negative in patients (right: −2.89°C, left: −2.91°C, mean: −2.90°C) than in controls (right: −2.36°C, left: −2.42°C, mean: −2.39°C) (*P* = 0.37 for mean values). Patients’ great toes were colder than ‘other’ (lesser) toes (right: −2.58°C, left: −2.63°C), although not significantly. In patients with SSc, finger and great toe temperature gradients were correlated (*r* = 0.406, ρ = 0.01).

**Conclusion:**

Our findings suggest that the great toe is the coldest in patients with SSc and that patients with the coldest fingers tend to have the coldest toes. Severe RP symptoms in the hands should prompt podiatry assessment and foot care education. Mobile phone thermography is a convenient tool for assessing the digital vasculature but first requires validation in larger studies with a longitudinal component.

Key messages

- Most patients with SSc experience RP in the feet; the great toe is most affected.
- Severities of RP in the feet and hands correlate, as assessed by thermography.
- Mobile phone thermography has potential as an inexpensive tool to assess the foot vasculature.

## Introduction

Studies of RP, including RP secondary to SSc, have predominantly assessed the hands and few have assessed the toe vasculature. The toes are, of course, much less ‘visible’ than the fingers and so patients tend to be less aware of colour changes in the toes [1]. However, many patients with SSc experience significant RP symptoms in the toes. Reported prevalences of RP in the toes are on the order of 70–90% [1–3], with some patients experiencing symptoms severe enough to warrant botulinum toxin injections [4]. Anecdotally, patients often report that the great (first) toe is most affected, in contrast with the situation in the hands where the first digit (the thumb) is the least affected digit [5, 6]. In patients with SSc, RP of the feet can progress (as in the fingers) to digital ulceration [2].

Ideally, studies of RP should therefore include assessment/measurement of vascular abnormalities not only in the fingers, but also in the toes. Infrared thermography offers a relatively simple means of assessing blood flow (albeit indirectly by measuring surface temperature) and has been applied extensively in clinical trials of RP of the fingers as a measure of treatment response [7]. The introduction of mobile phone thermography, which is portable and relatively low cost, brings the possibility of non-invasively assessing RP/digital vasculopathy of the toes as well as the fingers in an outpatient setting.

Our overall aim was to assess, in a pilot study, the toe vasculature in patients with SSc using mobile phone thermography, with specific objectives being to investigate the following hypotheses in patients with SSc: that the temperature gradient between the dorsum of the foot and toes (distal–dorsal difference [DDD]) is more ‘negative’ (toes cooler) than in healthy controls, that this temperature gradient is greatest along the first (great) toe and that in patients with SSc, the severity of thermographic abnormalities in the feet is associated with the severity of thermographic abnormalities in the hands.

## Methods

### Participants

Forty patients with SSc were recruited from the outpatient clinic at Salford Royal Hospital, a tertiary centre for SSc, and were subdivided into limited cutaneous and diffuse cutaneous subtypes on the basis of the extent of their skin involvement [8]. Twenty healthy controls were also recruited. All participants were ≥18 years of age. Patients were asked to rate the severity of the RP in their hands and feet over the past week (each on a separate scale of 0–10 points). They were also asked to rate, on a visual analogue scale (VAS; 0–100), the degree of functional limitation due to finger ulcers over the past week and their overall disease severity over the past week. The study was approved by the Yorkshire and Humber South Yorkshire Research Ethics Committee and also by the Health Research Authority and Health and Care Research Wales (REC reference: 19/YH/0048). All patients signed an informed consent.

### Thermography

**Figure 1.**
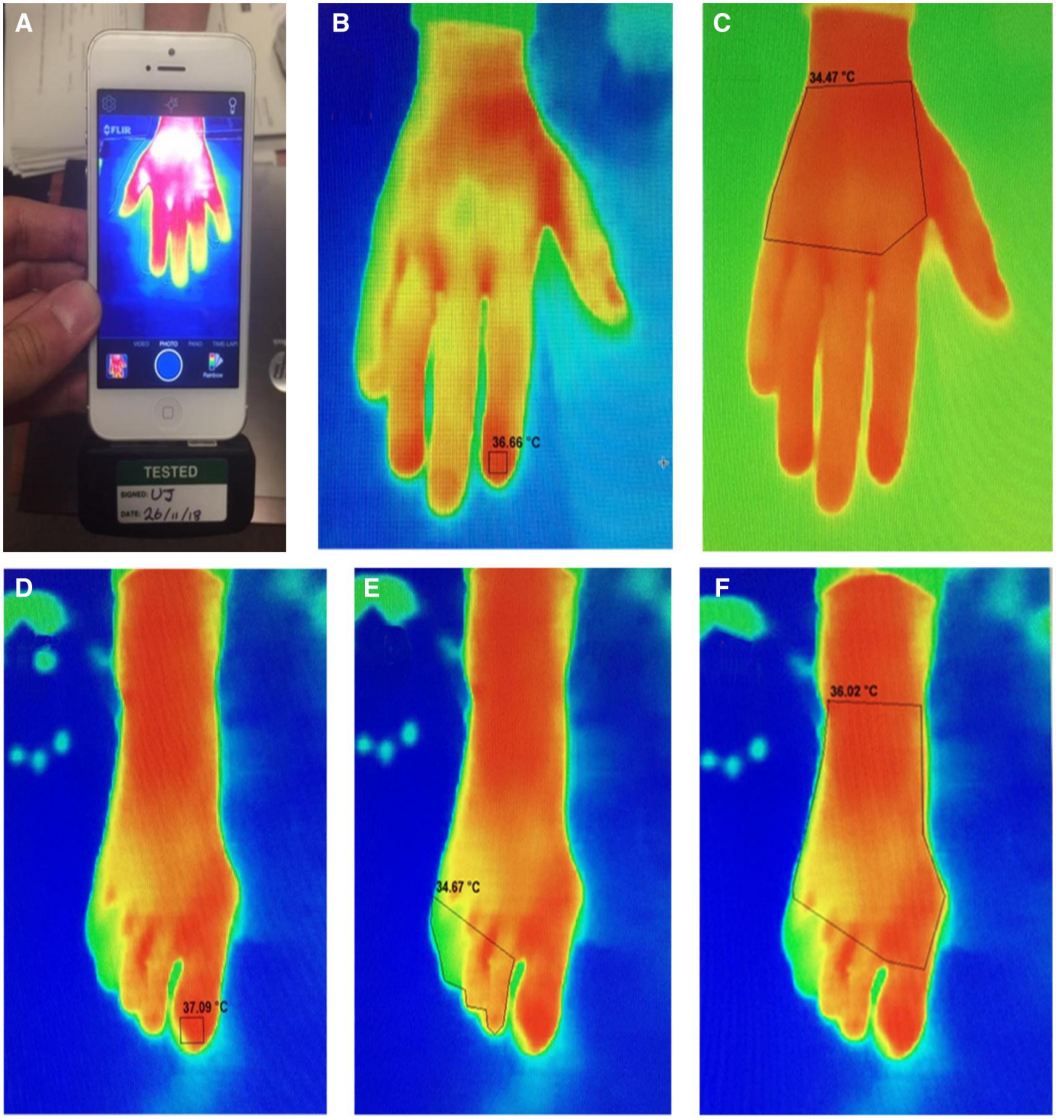
Mobile phone thermography **(A)** demonstrating how the thermal image is visualized on the phone with **(B–F)** the ROIs outlined on the right hand (B and C) and right foot (D–F)

After 5 minutes of acclimatization at room temperature, thermographic images of the dorsum of each hand and foot were captured using a Flir One thermal camera (Flir Systems, West Malling, UK) attached to an iPhone 4 (Apple, Cupertino, CA, USA) (Fig. 1A). The images of each subject were analysed by outlining regions of interest (ROI), as shown in Fig. 1B–F. In each hand, temperatures were measured (by thermography) at the dorsum and at the tips of the index, middle, ring and little finger (Fig. 1B, C). In each foot, temperatures were measured at the dorsum, the tip of the great toe and the other (lesser) toes (toes 2–5, combined into one measurement due to difficulty in distinguishing the tips of the lesser toes on a thermal image) (Fig. 1D–F). Based on these temperature data, the DDD [temperature of the tip of finger or toe(s) minus the temperature of the dorsum of the hand or foot] was calculated for the right and left index, middle, ring and little fingers; the right and left great toes; and the right and left lesser toes. From these values, the following measurements for each participant were derived: mean finger DDD (mean of the eight fingers), mean great toe DDD (mean of right and left great toes) and mean lesser toe DDD (mean of the right and left lesser toes). A negative value for the DDD indicates that the finger or toe temperature is lower than the temperature of the dorsum of the hand or foot.

### Statistical analysis

Demographic characteristics were found to be not normally distributed and hence are reported as medians and ranges. However, the thermography measurements were considered normal enough for parametric testing and were reported as mean (s.d.). Toe DDDs were compared between patients and controls using independent samples *t*-tests. Great toe and lesser toe DDDs were compared using paired samples *t*-tests. To look for an association between the severity of thermographic abnormality in the hands and feet, mean finger DDD was correlated with mean great toe DDD using Pearson product moment correlation. *P*-values <0.05 were considered significant. Statistical analysis was carried out using the Analysis ToolPak in Excel (version 2016; Microsoft, Redmond, WA, USA).

## Results

Of the 40 patients with SSc, 35 (87.5%) were female, median age was 61.5 years (range 24–79), and 4 (10%) were smokers. Of the 20 healthy controls, 15 (75%) were female, median age 31.5 years (range 21–58), and 2 (10%) were smokers. Of 39 patients with SSc (data missing for 1 patient), 26 (67%) reported RP of the feet. Of all 40 patients, 27 (67.5%) had limited cutaneous SSc and 13 (32.5%) had diffuse cutaneous SSc. Ten (25%) had had at least one previous admission for intravenous vasodilator therapy and 5 (12.5%) had had at least one digital debridement. The median RP severity score in the hands (*n* = 40) was 5.5 [interquartile range (IQR) 2–8] and in the feet (*n* = 34) it was 2.5 (IQR 0–5). The median VAS score (0–100) for functional impact of digital ulcers was 0 (IQR 0–40) and for overall disease severity it was 49 (IQR 24–81). Of the 40 patients, 6 had hypertension (1 of whom also had ischaemic heart disease) and 1 had diabetes. Thirty (75%) were on vasoactive therapies: 21 were on a calcium channel blocker, 11 on a phosphodiesterase inhibitor, 2 on an endothelin-1 receptor antagonist, 2 on an angiotensin II receptor antagonist and 6 on an angiotensin-converting enzyme inhibitor (12 were on more than one medication).

### Comparing patients with healthy controls

#### Great toe measurements

**Figure 2.**
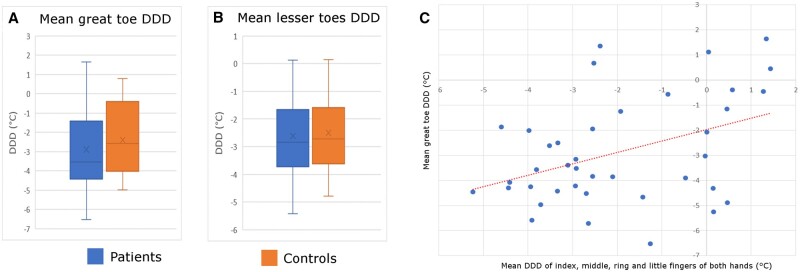
Thermography results. DDD of the mean of the right and left **(A)** great toes and **(B)** lesser toes in patients (blue) and healthy controls (brown). **(C)** Scatterplot of the mean finger DDD *vs* the mean great toe DDD

There were no significant differences in great toe DDDs between patients and controls, although there was a trend for the gradient to be ‘more negative’ [i.e. toe(s) cooler] in patients than in controls (Supplementary Table S1, available at *Rheumatology Advances in Practice* online). The mean right great toe DDD was −2.89°C (s.d. 2.21) in patients and −2.36°C (s.d. 2.16) in controls (*P* = 0.38). The mean left great toe DDD was −2.91°C (s.d. 2.04) in patients and −2.42°C (s.d. 1.91) in controls (*P* = 0.37). When considering the DDD of right and left great toes, the mean was −2.90°C (s.d. 2.07) in patients and −2.39°C (s.d. 1.98) in controls (*P* = 0.37) (Fig. 2A).

#### Lesser toe measurements

Lesser toe DDDs were similar in patients and controls (Supplementary Table S1, available at *Rheumatology Advances in Practice* online). The mean of the right lesser toes was −2.58°C (s.d. 1.53) in patients and −2.52°C (s.d. 1.37) in controls (*P* = 0.86). The mean of the left lesser toes was −2.63°C (s.d. 1.23) in patients and −2.47°C (s.d. 1.32) in controls (*P* = 0.65). When considering the DDD of the right and left lesser toes, the mean was −2.61°C (s.d. 1.34) in patients and −2.49°C (s.d. 1.33) in controls (*P* = 0.76) (Fig. 2B).

### Comparing temperature gradients along great toes *vs* lesser toes

#### Patients

Temperature gradients (toes colder) were greater along great toes than along lesser toes, although not significantly (Supplementary Table S1, available at *Rheumatology Advances in Practice* online). The mean was −2.89°C (s.d. 2.21) for the right great toe and −2.58°C (s.d. 1.53) for the right lesser toes (*P* = 0.11). The mean was −2.91°C (s.d. 2.04) for the left great toe and −2.63°C (s.d. 1.23) for the left lesser toes (*P* = 0.12).

#### Controls

Temperature gradients were similar along great toes and lesser toes (Supplementary Table S1, available at *Rheumatology Advances in Practice* online). The mean was −2.36°C (s.d. 2.16) for the right great toe and −2.52°C (s.d. 1.37) for the lesser toes (*P* = 0.54). The mean was −2.42°C (s.d. 1.91) for the left great toe and −2.47°C (s.d. 1.32) for the lesser toes (*P* = 0.82).

### Correlating thermographic findings in hands and feet

One patient was excluded from this correlation analysis because thermal images of both hands were not available. There was a positive correlation between finger and great toe DDDs (*r* = 0.406, ρ = 0.01) (Fig. 2C). This means that the colder the fingertips, the colder the toes.

## Discussion

The main findings of this pilot study were that, in patients with SSc, the great toe is the most affected (coldest) toe and that patients with the coldest fingers are those with the coldest toes. The study also demonstrated the feasibility of mobile phone thermography to assess perfusion of the hands and feet in an ambulatory setting. The great toe is the longest toe (in contrast to the thumb which is the shortest digit), which may contribute to its being the coldest.

Thermographic imaging has seldom been applied in studying the toe vasculature in patients with RP. Lim *et al*. [9] undertook a study of 33 patients with primary RP, 24 with secondary RP (of whom 10 had SSc) and 146 healthy controls, focusing on the great toe. Findings included that the great toe DDD could help to discriminate between patients and controls, consistent with our finding that patients’ great toes were cooler than controls’: a gradient of >3.11°C (toe cooler) had a sensitivity and specificity of 73% and 66%, respectively [9]. The great toe temperature gradient was higher in Lim *et al*.’s study [9] (dominant side 3.63°C, non-dominant side 3.79°C) than in ours, perhaps reflecting different patient populations, different placement of the ROIs, different thermography equipment and the fact that Lim *et al*.’s study included full acclimatization for 30 min in a temperature-controlled room.

Although it might seem intuitive that in patients with SSc the degree of vascular abnormality in the fingers correlates with that in the toes, our study provides confirmation. Foot problems can be a major issue in patients with SSc [2, 3, 10] but are often overlooked. An impaired blood supply will predispose to toe ulcers, and the great toe may be especially vulnerable to pressure effects if a hallux valgus deformity is present. A ‘take-home’ message from our findings is that clinicians should be especially vigilant in examining the feet of patients with severe RP of the fingers.

Although mobile phone thermography gives less accurate readings than ‘standard’ (and more expensive) thermography (±5% from the ‘real’ temperature, as opposed to ±2% with standard thermography [11, 12]), the two methods correlate [13], and mobile phone thermography has the advantage of simplicity and being much less expensive. Mobile phone thermography therefore deserves further validation as a technique that may be helpful in diagnosis, in studying pathophysiology and treatment response and as a predictor of digital ulceration. It has been suggested that the degree of thermographic abnormality in the hands predicts finger ulcer risk in patients with SSc [14]. If this is true as well for mobile phone thermography (and in toes as well as fingers), then this could provide the clinician with a simple tool for stratifying risk not only in SSc, but in other vascular conditions, e.g. diabetes [15, 16].

Our study had limitations, mainly the small numbers involved, which were, however, appropriate for a pilot study. Also, controls were younger than patients, although this was not relevant to the main findings of the study, which related to within-patient comparisons.

In conclusion, mobile phone thermography demonstrated, in patients with SSc, the relationship between vascular abnormalities in the hands and feet, and how the great toe is the toe most affected by vascular compromise. Validation studies in larger numbers of patients (including community-based studies) and longitudinal studies examining risk factors for progression are now required to establish the clinical utility of mobile phone thermography in the assessment and prevention of foot problems in patients with SS, and potentially in the assessment of other causes of secondary RP affecting the feet, such as vibration-induced injury [17].

## Supplementary Material

rkae068_Supplementary_Data

## Data Availability

The sponsor will share de-identified individual participant data collected during the study with researchers who provide a methodologically sound proposal.
