# Supplementary material for: Mobile phone thermography of the toes in patients with systemic sclerosis—a pilot study
Source: Rheumatol Adv Pract. 2024 May 22;8(3):rkae068. doi: 10.1093/rap/rkae068 (PMC11160323; doi:10.1093/rap/rkae068)
Supplement: rkae068_Supplementary_Data [file rkae068_supplementary_data.docx]

**Supplementary Table S1. Thermography measurements of the toes in patients and healthy controls.**

|  | Patients (n= 40) | | | Healthy controls (n=20) | | | P value for difference (patients vs controls) | | |
| --- | --- | --- | --- | --- | --- | --- | --- | --- | --- |
|  | Right | Left | Mean | Right | Left | Mean | Right | Left | Mean |
| Great toe DDD (^O^C) | -2.89 ± 2.21 | -2.91 ± 2.04 | -2.90 ± 2.07 | -2.36 ± 2.16 | -2.42 ± 1.91 | -2.39 ± 1.98 | 0.38 | 0.37 | 0.37 |
| Lesser toes DDD (^O^C) | -2.58 ± 1.53 | -2.63 ± 1.23 | -2.61 ± 1.34 | -2.52 ± 1.37 | -2.47 ± 1.32 | -2.49 ± 1.33 | 0.86 | 0.65 | 0.76 |
| P value for difference (great toe vs lesser toes) | 0.11 | 0.12 | 0.46 | 0.54 | 0.82 | 0.85 | - | - | - |

Values are mean (standard deviation). DDD: Distal-dorsal difference.
